# The Effect of Vitamin D3 Alone and Mixed With IFN-γ on Tachyzoites of *Toxoplasma gondii* (RH Strain) Proliferation and Nitric Oxide (NO) Production in Infected Macrophages of BALB/C Mice

**Published:** 2010-09

**Authors:** F Ghaffarifar, M Abdolah Pour, Z Sharifi, A Dalimi Asl, E Al-Kawaz

**Affiliations:** 1 Department of Parasitology, Tarbiat Modarres University, Tehran, Iran; 2 Organization of Blood Transfusion, Research Center of Virology, Tehran, Iran; 3 Department of Public Health, College of Medical and Health Technology, Baghdad, Iraq

**Keywords:** *Toxoplasma gondii*, Vitamin D3, Nitric Oxide, IFN-γ, Macrophages, Mice

## Abstract

**Introduction:**

*Toxoplasma gondii* is an obligatory interacelullar parasite that infects nucleated cells in its intermediate hosts. The aim of the present study was to determine the effect of vitamin D3 on the multiplication of *T. gondii* in peritoneal macrophage of Balb/c mice and nitric oxide production by macrophages.

**Methods:**

According to usage of vitamin D3 (one dose or seven doses) and INFγ in vitro and in vivo, this study was divided into four experiments. In all experiments, the macrophages were collected from peritoneum and cultured in RPMI-1640. Then the supernatants were collected after 24 h and their nitric oxide was measure. After 96 h, the macrophages were collected and stained and the number of tachyzoites was measured.

**Results:**

The first experiment (the mice were infected with tachyzoites and after 2 h, got one dose vitamin D3 intraperitonealy) showed the best results. The mean of tachyzoites per macrophages was 2.37, and mean±SD of nitric oxide was 187.8±9.

**Discussion:**

High-level production of nitric oxide may be related to the only one injection of vitamin D3. The injection in long time might suppress the immune system.

## Introduction

*Toxoplasma gondii* is an obligatory intracellular protozoan parasite that infects all mammalian cells. Human infection is generally asymptomatic and self-limiting in immunocompetent hosts. The parasites are encysted in the brain and muscle and the infected individuals chronically develop life-long protective immunity against reinfection (1,2). In contrast, in immunocompromised individuals, toxoplasmosis represents one of the major opportunistic infections (3,4). It is most often due to reactivation of the latent infection and may result in toxoplasmic encephalitis (3,4). Inducing synthesis of nitric oxide (NO) is an important microbicidal mechanism of IFN-γ activated murine macrophages to restrict interacelullar replication of *T. gondii* (5,6). Inhibition of NO production in mice could led to progressive toxoplasmic encephalitis and chronic ocular toxoplasmosis (7,8).

The role of vitamin D in the regulation of calcium (Ca) and phosphorus metabolism is well-established (9). There are noncalcemic roles of vitamin D, including its role in the immune system (10). Receptors for 1,25-dihydroxy vitamin D3 (VDR) have been found in human peripheral blood monocytes and in the active lymphocytes of both rat and human (11,12). In addition to *in vitro* activated lymphocytes, *in vivo* expression of the VDR occurs in peripheral blood lymphocytes of rheumatoid arthritis patients (13), in alveolar lymphocytes from the patients with pulmonary granuloma diseases such as sarcoidosis and tuberculosis (14). On the other hand, decreased concentrations of VDR are found in peripheral blood mononuclear cells in the patients with x-linked hypophosphatemic rickets (15). Furthermore, 1,25-(OH)2D3 exerts an antiproliferative action on peripheral blood mononuclear cells (16) and decreases interleukin-2 (IL-2) production by phytohemaglutinin activated human peripheral mononuclear cells (17). A major advance occurred with this demonstration that 1, 25-(OH)2D3 can prevent or markedly suppress such autoimmune diseases as experimental autoimmune encephalomyelitis (18,19), experimental arthritis in mice (20), and Type I diabetes in NOD mice (21). Further, 1, 25-(OH) 2D3 can prolong transplanted allograft survival equal to or superior to cyclosporin (22), demonstrating the importance of 1,25-(OH)2D3 in the immune system. 1,25-(OH)2D3 appears to function largely, if not exclusively, through a nuclear receptor (VDR) (23), which is a member of the steroid/thyroid hormoneactivated transcription factor family and is believed to act via binding as a heterodimer to vitamin D-responsive elements found in the promoter of target genes (24). Although nongenomic actions of 1,25-(OH)2D3 have been championed (25), so far its physiologic significance remains to be established. The results of a survey by Rokett et al. showed that 1, 25 dihydroxy vitamin D3 may increase production of nitric oxide (26).

In some groups of African and Americans, has been observed that low levels of the active form of vitamin D3 that often occur in this population correlate with increased susceptibility to infection. Vitamin D3 may be related in the immune response to tuberculosis too. 1,25(OH)2D3 is known to increase the phagocytic potential of macrophages infected with live *Mycobacterium tuberculosis* in normal healthy subjects and decrease the lymphoproliferative response in pulmonary tuberculosis patients (27).

The present study was conducted to evaluate the effect of vitamin D3 and IFN-γ alone or in combination on the growth rate and replication of *T. gondii* in macrophages.

## Materials and Methods

### Parasites

Tachyzoites of *T. gondii* (RH strain) were injected intraperitonealy in the mice. The mice were killed after 3 days and the tachyzoites were collected from their peritoneum.

### Animals

We used 84 inbred female mice (Balb/c) with the age of 6-8 weeks and the weight of 18-20g. The mice were divided into four experiments and each experiment consisted of seven groups of mice (three mice in each group).

### Vitamin D3 and IFN-γ

We obtained the materials from Sigma Chemical Co. Ethanol 95% was applied as a solvent for vitamin D3. Evaluation of the effect of vitamin D3 and IFN-γ on the proliferation of *T. gondii* and NO production by the infected macrophages was performed in four separated experiments including:

In the first experiment, vitamin D3 and 1.0 ×10^6^ *T. gondii* tachyzoites were intraperitonealy injected 24 hrs before macrophages collection in the mice, (the interval between vitamin D3 and tachyzoites injection was 2 h).

In the second experiment, vitamin D3 was intraperitonealy injected for one week (every day for seven days),and then infected by1.0 ×10^6^ *T. gondii* tachyzoites (the interval between the last injection of vitamin D3 and tachyzoites injection was 2 h). In both experiments, 24 h after tachyzoites injection, the macrophages were collected and cultured in triplicate in 24-well cell culture plates (Nunc) (2×10^6^ macrophages/well).

In the third experiment, the mice were infected by 1.0 ×10^6^ *T. gondii* tachyzoites. The infected macrophages were collected 24 h after and cultured in triplicate in 24-well cell culture plates (Nunc) (2×10^6^ macrophages/well). Then vitamins D3 were added to the macrophage culture.

In the fourth experiment, vitamin D3 was intraperitonealy injected to the mice, the macrophages separated from the peritoneum 24h after and cultured in 24-well cell culture plates (2×10^6^ macrophages/well). Then1.0 ×10^6^ *T. gondii* tachyzoites were added to cultured macrophages.

### Grouping for each experiment

Each experiment consisted of seven groups of mice.

1. First control group, the macrophages were infected with 1.0 ×10^6^ *T. gondii* tachyzoites.
2. Second control group, the macrophages were infected with 1.0 ×10^6^ *T. gondii* tachyzoites and ethanol was added the as the solvent of vitamin D3.
3. The group that before infection with 1.0 ×10^6^ *T. gondii* tachyzoites got the vitamin D3, and then IFN-γ added to cultured macrophages.
4. The group that before infection with 1.0 ×10^6^ *T. gondii* tachyzoites got the vitamin D3, without the addition of IFN-γ.
5. The group that infected with 1.0 ×10^6^ *T. gondii* tachyzoites, then IFN-γ added to cultured macrophages without vitamin D3.
6. The group that infected with 1.0 ×10^6^ *T. gondii* tachyzoites, then NMMA (*N* ^G^-methyl-l-arginine) 1mM was added (as negative control).
7. The group that infected with 1.0 ×10^6^ *T. gondii* tachyzoites, then SNAP (*S*-nitroso-acetylpenicillamin) 1mM was added (as positive control).

### Nitric Oxide (NO) measurement

In aqueous solution, nitric oxide rapidly degrades to nitrate and nitrite, for Spectrophotometric quantitation of nitrite using Griess Reagent is sensitive.

### Griess reagent

Griess reagent contains of two solution, sulfanilamide solution and naphthylethylenediamine dihydrochloride solution that prepared separately, stored at 4°C, and protected from light. The Griess reagent is mix of 1 part 0.1% naphthylethylenediamine dihydrochloride in distilled water plus 1 part 1% sulfanilamide in 5% H_3_PO_4_.

For NO measurement, the supernatants were collected daily and compared with eight nitrite standard concentrations (0 to 35 µM). For nitrite standard, we prepared sodium nitrite NaNO_2_ (MW=69) 1000 µM as stock solution, then prepared 8 standards concentration (0, 5, 10, 15, 20, 25, 30 and 35 µM).

One hundred µl of each of the standards and the samples (supernatants of the cell cultures) was added into the wells of a 96-well plate in duplicates. Then, 100 µl of Griess reagent was added to each well and the plate was incubated at room temperature for 10 min. The absorbance was read at 550 nm on a plate reader (28).

### Statistical analysis

For analysis of the differences between the mean of parasites in macrophages and NO production among the different groups Anova-oneway test was used.

## Results

The results indicated that vitamin D3 could affect *T. gondii* proliferation and NO production by infected macrophages. The best results for the inhibition of tachyzoites proliferation were obtained in the fourth and the first experiments using IFN- γ (100 IU) and vitamin D3 (1000 IU) (2.03±0.1875,2.37± 0.1901 tachyzoites per macrophages respectively) and the difference with the control group was significant. For NO production by the infected macrophages, the best results were obtained in the same groups (191.5±9.62, 187.8 ±0.9821, respectively). The results for other groups are shown in Tables 1 and 2.

**Table 1:** Mean±SD of tachyzoites per macrophages after 96 h of culture in cell culture media

Groups Experiments	Control Group1 Mean±SD	Solvent (ethanol 95) Group2 Mean±SD	IFN- γ (100 IU) & Vitamin D3 (1000 IU) Group3 Mean±SD	Vitamin D3 (1000 IU) Group4 Mean±SD	IFN- γ (100 IU) Group5 Mean±SD
**First**	3.01±o.14	2.93±0.16	*2.37±0.19	*2.49±0.19	*2.6±0.2
**Second**	3.15±0.12	3.03±0.16	*2.58±0.13	2.74±0.16	*2.5±0.15
**Third**	3.05±0.15	3.04±0.14	*2.69±0.2	2.82±0.17	*2.57±0.16
**Fourth**	3.16±0.14	3±0.14	*2.03±0.19	*2.39±0.19	*2.59±0.2

SD=standard deviation INF-γ=interferon gamma IU=International Unit

* Significan differences *P*≤0.05

**Table 2:** Mean±SD of nitrite (M/ml) produced by macrophages after 24 h of culture in cell culture media

Groups Experiments	Control Group1 Mean±SD	Solvent (ethanol 95) Group2 Mean±SD	IFN- γ (100 IU) & Vitamin D3 (1000 IU) Group3 Mean±SD	Vitamin D3 (1000 IU) Group4 Mean±SD	IFN- γ (100 IU) Group5 Mean±SD
**First**	109 ± 8.02	108.2±12.45	*187.8±9.82	*165±11.30	*146±7.22
**Second**	108±9.46	108.9±6.93	*136.2±10.21	121.2±6.68	*139.5±5.76
**Third**	109.6±7.35	108.2± 4.96	*146.9±9.62	*139±7.01	*146±4.93
**Fourth**	109±7.03	108.6±4.26	*191.5±9.62	*166±7.01	*146.2±5.60

SD=standard deviation INF-γ=interferon gamma IU=International Unit

* Significant differences *P*≤0.05

In the second experiment, the effect of using vitamin D3(1000 IU) had no significant differences with the control groups, but using IFN- γ(100 IU) and vitamin D3(1000 IU) or IFN- γ(100 IU), showed significant differences with the control groups (*P*<0.05).

For NO production by the infected macrophages, in the second experiment, there were significant differences among groups with IFN- γ(100 IU) and IFN- γ(100 IU) plus vitamin D3(1000 IU) with control groups (*P*<0.05). In this experiment, there was not any significant differences among vitamin D3 group (1000 IU) with control groups (*P*>0.05).

The results for NMMA in group 6, and SNAP in group 7 was approximately the same in both nitric oxide production and tachyzoites proliferation in four experiments.

## Discussion

Epidemiological studies have linked vitamin D deficiency to increased rates of cancer, as well as autoimmune and infectious diseases (29).

Insufficient vitamin D is related to some infectious disease such as tuberculosis. Associations between vitamin D deficiency and TB susceptibility were described over 20 years ago (30,31).

The activated form of vitamin D on calcium and bone metabolism has been described. In vitro, but also in vivo, 1,25(OH)_2_D_3_ has potent effects on cell proliferation and cell differentiation in normal cell types as well as in malignant cell types. Receptors for 1,25(OH)_2_D_3_ have been found in different immune cells such as the cells of the monocyte to macrophage lineage, (32) activated T and B lymphocytes. In vitro, clear effects can be seen, including the induction of monocyte differentiation and the inhibition of antigen presentation by antigen-presenting cells, inhibition of T-cell proliferation and cytokine production, and inhibition of secretion of antibodies by B-cells. These in vitro effects are reflected in vivo by a potential to prevent autoimmune diseases in different experimental models, and to prolong graft survival (33–34).

A major problem with the *in vivo* use of 1,25(OH)_2_D_3_ is the fact that the doses needed to see non-classical effect such as the immune effects are high, resulting in severe hypercalcaemia and accelerated bone remodeling. Due to the flexible structure of 1,25(OH)_2_D_3_, however, chemists have succeeded in synthesising analogues of the molecule, some of which share the mother molecules’ effects on the immune system, but not its effects on calcium or bone metabolism (35–36).

In the previous study we were found that in the wells treated with IFN-γ, replication rates of *T. gondii* were decreased significantly 72h post inoculation in comparison with control group (*P*<0.05). There was no significant difference among different groups in NO production (37).

In this study, the results in the first experiment showed that vitamin D3 had strong effect on the inhibition of tachyzoites proliferation and on the increase of NO production, and that the effect of this vitamin was more than IFN- γ. When IFN- γ was added to this vitamin, it showed potent effects on the inhibition of tachyzoites proliferation and on the increase of NO production. The results also showed that the use of IFN- γ and vitamin D3 can reinforcement each other.

In the second experiment, the effect of using vitamin D3(1000 IU) had no significant differences with the control groups, but using IFN- γ(100 IU) and vitamin D3(1000 IU) plus IFN- γ(100 IU) had significant differences with the control groups (*P*<0.05). In this experiment, the vitamin D3 did not have any considerable effect on the inhibition of tachyzoites proliferation and NO production and the differences were not significant. In the group that used vitamin D3 (1000 IU) plus IFN- γ(100 IU), the effect was related more to IFN- γ and no to the vitamin D3. In this experiment, the results showed that when vitamin D3 was added to macrophage culture (in-vitro), could less be The authors declare that they have no conflicts of interest.-verted to the effective form (1,25(OH)_2_D_3_). 1,25-dihydroxy-cholecalciferol is the most powerful metabolite of vitamin D3 and the highest effect of vitamin D3 is related to this metabolite.

In the third experiment, where the mice got vitamin D3 (1000 IU) for one week, the effect on the inhibition of *T. gondii* tachyzoites and NO production was less than in the first experiment which vitamin D3 was used only once. These results indicated that the injection of vitamin D3 every day for one week might have more suppressive effect on the immune system.

In the fourth experiment, the results were better than in other experiments and indicated that the injection of vitamin D3, 24 h before adding *T. gondii* was more effective than other experiments.

The probable reason of differences between the four experiments could be the influence of 1- α hydroxylaze *in vivo* that affected vitamin D3 and changed it into activated form of 1,25 dihydroxy vitamin D3. When vitamin D3 was added in vitro, it was less changed into its activated form of 1,25 dihydroxy vitamin D3 because there was not any 1- α hydroxylaze enzyme *in vitro* condition.

In conclusion, the results indicated that, high-level production of nitric oxide might be related to one injection of vitamin D3 in the first and fourth experiments.
